# Glucose may be biotransformed and then react with alcohol, fats, endocannabinoids, or various amino acids to form dopamine or norepinephrine *in vivo*

**DOI:** 10.3389/fphar.2025.1715092

**Published:** 2025-11-06

**Authors:** Paul J. Fitzgerald

**Affiliations:** Independent Researcher, West Lafayette, IN, United States

**Keywords:** sucrose, serine, alanine, aspartate, phosphatidylethanolamine, stable isotope, liquid chromatography, mass spectrometry

## Introduction

**FIGURE 1:**
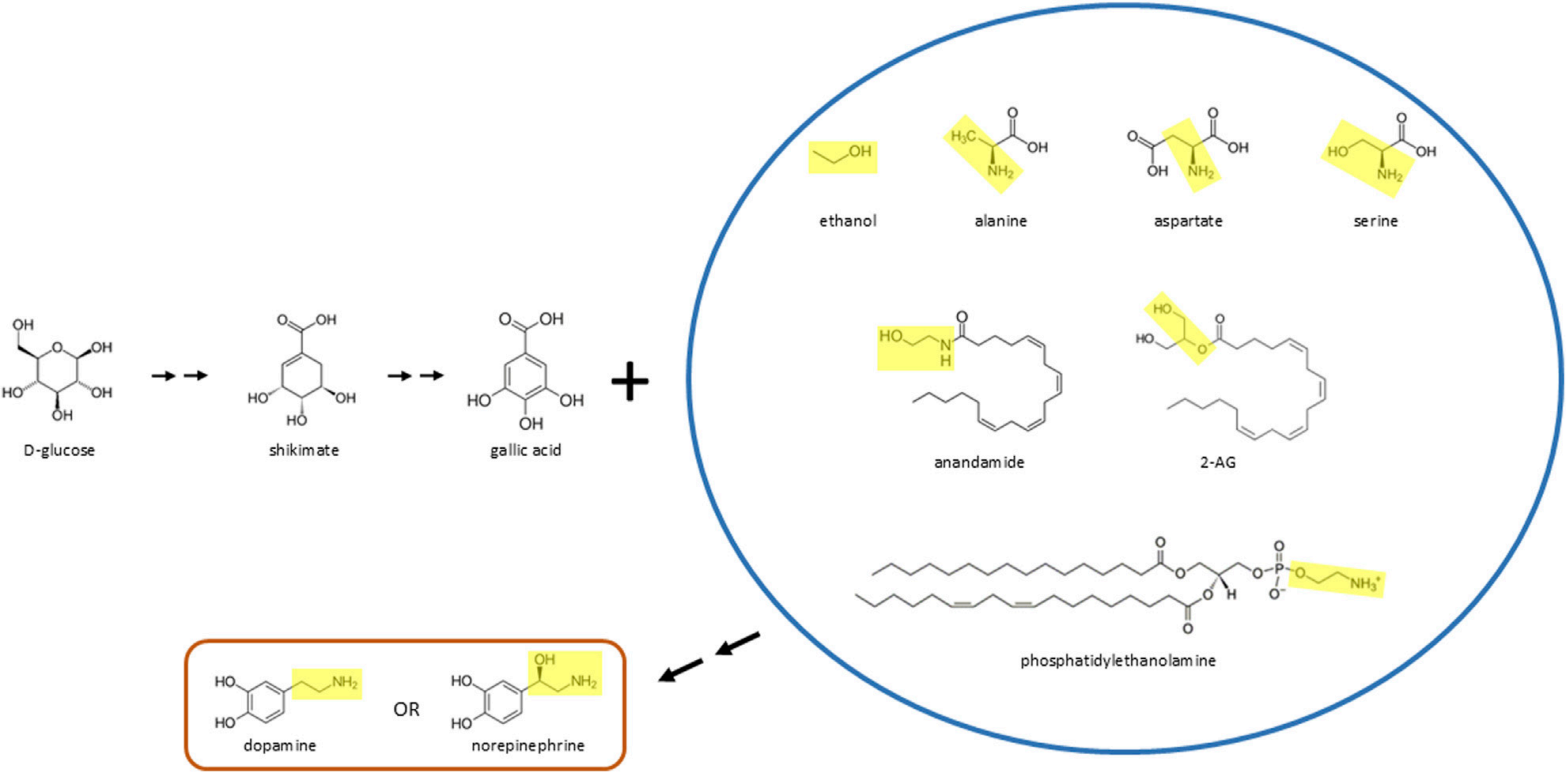
D-glucose may be biotransformed and then react with various molecules to form catecholamines *in vivo*. In this scenario, D-glucose is converted to gallic acid (or a similar phenylic molecule) and then reacts with ethanol, various amino acids (alanine, aspartate, serine), endocannabinoids (anandamide, 2-AG), or fats (phosphatidylethanolamine or related molecules) to form dopamine or norepinephrine. The yellow boxes inside the blue circle highlight the moiety, in many cases ethylamine or ethanolamine, that reacts with gallic acid (which is decarboxylated) to form dopamine or norepinephrine, which can then lead to formation of epinephrine through the canonical molecular pathway described over 70 years ago. Double arrows indicate two or more chemical reactions.

A previous publication suggested that D-glucose can be biotransformed to yield a broad range of neurotransmitters, including the catecholamines dopamine, norepinephrine, and epinephrine (Fitzgerald, 2020). That paper also proposed that various phytochemicals which bear a benzene ring structure can react with ethanol, fats, and the amino acid serine to yield dopamine. In that scenario, ethanol, fats, and serine contribute the two hydrocarbon (ethylamine) side chain to form dopamine. The current publication suggests an additional way in which these molecules and others interact to form catecholamines: D-glucose may be converted to the phenolic molecule gallic acid, which then reacts with ethanol, fats, endocannabinoids, or various amino acids (alanine, aspartate, serine) to form dopamine or norepinephrine (Figure 1) (Fitzgerald, 2025). Afterward, dopamine or norepinephrine can be transformed to create epinephrine through the canonical molecular pathway described over 70 years ago (Holtz, 1939; Blaschko, 1942; Bulbring, 1949). Thus, the current publication expands upon these previous ideas, and suggests that D-glucose is transformed to the benzene ring containing molecule gallic acid, which can then react with a broad range of other molecules that attach to the ring and help form the side chain of catecholamines. As noted below, there is already evidence for conversion of D-glucose to gallic acid in various non-mammalian organisms. Also, consistent with bioconversion of gallic acid to norepinephrine, gallic acid has anti-inflammatory properties (Recart et al., 2025), as does norepinephrine (Thoppil et al., 2023).

## Proposed enzymatic reactions

Conversion of glucose to gallic acid (via shikimate) has already been demonstrated in plants and fungi (Dewick and Haslam, 1969; Werner et al., 1997), but here it is suggested that this pathway exists in animals as well, possibly in bacteria of the microbiome that could supply neurotransmitters for the body (Chen et al., 2021). This proposed pathway may also yield phenylic (i.e., benzene ring containing) molecules related to gallic acid that react with the molecules inside the blue circle shown in Figure 1 to form catecholamines. (The reactions described in this paper may be a separate set of biosynthetic pathways from those involving D-glucose described in the original publication (Fitzgerald, 2020)). Gallic acid is thought to be present in humans largely through dietary consumption of various plant materials. Dietary, consumed gallic acid can modify and be metabolized by bacteria of the gut microbiome of humans and rodents (Marín et al., 2015; Esteban-Torres et al., 2018; Peng et al., 2024; Jian et al., 2025). Also, it is known that fecal samples of the human microbiome can metabolize various dietary phytochemicals to form gallic acid (Marín et al., 2015). This latter finding is consistent with the possibility that other molecular pathways exist for synthesizing gallic acid in the human microbiome, such as a pathway beginning with D-glucose.

As shown in Figure 1, the yellow moieties of the various molecules within the blue circle would combine with gallic acid to form the two hydrocarbon side chain present within dopamine or norepinephrine. In forming this side chain, the yellow moieties may need to be transformed, such as through decarboxylation or conversion of a hydroxyl group to an amine, depending on the parent molecule. These modifications would affect whether dopamine instead of norepinephrine is initially formed. Whereas hydroxyl to amine conversions may be widely known in chemistry applications, another possibility is that amines can also be converted to hydroxyl groups *in vivo*, which has been described in non-biological systems (Cheong et al., 2019). One possibility is that the yellow moieties shown in Figure 1 may also participate in biosynthesis of the benzene ring structure of newly forming catecholamines, possibly after these moieties are transformed to ethanol or ethanol-like molecules (Fitzgerald et al., 2025). Also, in the reactions described in this paragraph, the gallic acid molecule would need to be enzymatically decarboxylated and have one of its three hydroxyl groups removed, to form the catecholamines described here.

## Potential implications of bioconversion

If these novel pathways exist in a range of organisms, including humans, it may suggest that individuals with a high “set point” for one or more of these three catecholamines, may be inclined to consume sucrose (a disaccharide that contains the monosaccharides glucose and fructose), as well as fats, alcohol, and perhaps other dietary factors, to maintain elevated levels of these catecholamines (Fitzgerald, 2012; 2020). In such individuals, elevated norepinephrine or epinephrine release may suppress pancreatic insulin release, thereby increasing the systemic glucose level and allowing more glucose molecules to be converted to catecholamines throughout the body. This could promote glycolysis in a number of cell types, including in the context of the Warburg effect in cancer and perhaps other diseases. Lactic acid, whose production is enhanced during physical exercise, is a byproduct of glycolysis. Since physical exercise also enhances acute systemic release of norepinephrine and epinephrine (Jezova et al., 1985; Dimitrov et al., 2017), one possibility is that lactic acid, if it can be decarboxylated to yield ethanol, is used as a feedback mechanism to replenish depleted cellular stores of these two catecholamines through mechanisms described in this publication (and possibly other mechanisms). In *Drosophila*, glycolysis can produce alanine, which is one of the amino acids that is proposed here as a building block for catecholamines (Rabah et al., 2023).

Advancing age may also be associated with an elevated set point for norepinephrine, as sympathetic nervous system tone may become more prominent than parasympathetic tone as we age (Lee et al., 2004). Likewise, if psychological stress exposure ends up depleting the levels of the “stress hormones” norepinephrine and epinephrine (as well as dopamine), the organism may preferentially consume sucrose, fats, alcohol, and other dietary entities to replenish the depleted levels (Fitzgerald, 2012; 2020). The novel pathways described here may represent yet another way for the body to produce these important catecholaminergic molecules and assure their adequate supply. If these pathways exist *in vivo*, the enzymes catalyzing the various reactions would need to be identified and could be druggable targets for particular neuropsychiatric, neurologic, or metabolic disorders. The anatomical locations and cell types within the brain and in the periphery, such as the adrenal glands, where these putative reactions take place, also remain to be identified. One possibility is that if there is a novel biosynthetic pathway that converts glucose to norepinephrine or epinephrine, it may contribute to counteracting the deleterious effects of psychosocial stress that have been demonstrated in rodents (Conoscenti et al., 2017; Plumb et al., 2021). However, glucose consumption after marked psychosocial stress could instead restore metabolic homeostasis through mechanisms involving adenosine, corticosterone, or growth hormone, for example (Conoscenti et al., 2017; Plumb et al., 2021). Alternatively, therapeutic properties of glucose may be due to the rewarding value of a sweet drink, although other sweet substances such as saccharine and fructose do not seem to show these effects (Conoscenti et al., 2017; Plumb et al., 2021). But consistent with a role for glucose in biosynthesis of catecholamines, mice chronically fed a high sucrose diet indeed exhibit increased adrenal weight, as well as increased plasma norepinephrine and epinephrine (but not dopamine) (Miyata et al., 2025).

## Testing for bioconversion

Safe and relatively simple stable isotope (i.e., non-radioactive) biochemical experiments can be carried out to test whether the pathways described in this paper exist in animals and even humans (Fitzgerald, 2021). The basic idea is to label D-glucose with carbon 13 (or deuterium) (MilliporeSigma; D-glucose-^13^C_6_; catalog # 389374) for some of its atoms, also label the yellow moiety in one of the molecules shown inside the blue circle in Figure 1 (such as ethanol) with carbon 13 (MilliporeSigma; ethanol-1-13C; catalog # 324523) or deuterium, and then inject or infuse (intraperitoneally, subcutaneously, intravenously, or intracerebroventricularly) both labeled molecules into the model organism such as a mouse. Instead of ethanol, the following other molecules could be administered (as shown in Figure 1): alanine (MilliporeSigma; D,L-alanine-2,3,3,3-d4; catalog # 488917), aspartate (MilliporeSigma; D,L-aspartic acid-2,3,3-d3; catalog # 589667), serine (MilliporeSigma; D,L-serine-2,3,3-d3; catalog # 688436), anandamide (could be custom synthesized by various companies), 2-AG (MedChemExpress; 2-arachidonoylglycerol-d5; catalog # HY-W011051S1), or phosphatidylethanolamine (BOC Sciences, Shirley, NY). After a time delay of minutes to hours or up to a day or two, collect brain (possibly with microdialysis) with brainstem intact (where the noradrenergic locus coeruleus is located), blood plasma, or urine samples, and then use liquid chromatography-mass spectrometry to determine if one or more of the labeled atoms from both D-glucose and ethanol have been incorporated into new dopamine, norepinephrine, or epinephrine molecules. In this scenario, control animals would receive unlabeled carbon 12 (or normal hydrogen) D-glucose and unlabeled ethanol, to control for injection stress and acute intoxication. The liquid chromatography-mass spectrometry results could then be compared between the experimental and control animals, with perhaps at least ten animals in each group. An additional control group could receive labeled carbon 13 D-glucose and unlabeled carbon 12 ethanol, where a separate control group could get carbon 12 D-glucose and carbon 13 ethanol. A final control group could just receive two saline administrations.

In a separate experiment, stable isotope labeled gallic acid (LGC Standards, United Kingdom; gallic acid-d_2_; catalog # TRC-G188902-10 MG) could also be administered to mice to test the veracity of the proposed pathway, and whether it is converted to labeled catecholamines. Liquid chromatography-mass spectrometry could also be used to test whether stable isotope labeled D-glucose is converted to labeled gallic acid in blood, urine, or fecal samples of rodents or possibly humans. However, if gallic acid is a fleeting intermediate molecule in the bioconversion of D-glucose to catecholamines, it may be difficult to detect. Also, the suggestion above that lactic acid (MilliporeSigma; L-lactic acid-13C_3_; catalog # 746258) may be bioconverted to ethanol and eventually catecholamines, while controversial, should be amenable to testing with liquid chromatography-mass spectrometry. Consistent with decarboxylation to form ethanol from lactic acid, it is known that other enzymes *in vivo* are capable of decarboxylation, such as aromatic L-amino-acid decarboxylase (Rizzi et al., 2022).

## Conclusion

This publication has proposed that there are novel biosynthetic pathways in which D-glucose is transformed to gallic acid (via shikimate), and then reacts with portions of various other molecules (ethanol, endocannabinoids, various amino acids, and fats) to form the catecholamines dopamine and norepinephrine. It is suggested that these enzymatic pathways (or similar ones) exist in a wide range of organisms, not limited to mammals. If so, the existence of these pathways would alter our understanding of catecholamine physiology, and could eventually suggest druggable targets for psychiatric, neurologic, and metabolic disorders. Their existence can be probed with straightforward analytical chemistry experiments that combine stable isotope pharmacology with liquid chromatography-mass spectrometry.
